# Clinical Outcomes Associated with Endocervical Glandular Involvement in Patients with Cervical Intraepithelial Neoplasia III

**DOI:** 10.3390/jcm11112996

**Published:** 2022-05-25

**Authors:** Nae Ry Kim, Zee Hae Baek, A Jin Lee, Eun Jung Yang, Yung-Taek Ouh, Mi Kyung Kim, Seung-Hyuk Shim, Sun Joo Lee, Tae Jin Kim, Kyeong A So

**Affiliations:** 1 Department of Obstetrics and Gynecology, Konkuk University School of Medicine, Seoul 05030, Korea; kimnael77@gmail.com (N.R.K.); 20210090@kuh.ac.kr (Z.H.B.); 20170050@kuh.ac.kr (A.J.L.); 20200179@kuh.ac.kr (E.J.Y.); 20130131@kuh.ac.kr (S.-H.S.); 20050073@kuh.ac.kr (S.J.L.); 20190002@kuh.ac.kr (T.J.K.); 2 Department of Obstetrics and Gynecology, Graduate School of Medicine, Kangwon National University, Kangwon 24341, Korea; oytjjang@gmail.com; 3 Department of Obstetrics and Gynecology, Ewha Womans University Mokdong Hospital, Seoul 07985, Korea; asterik79@gmail.com

**Keywords:** cervical intraepithelial neoplasia, conization, endocervical glandular involvement, recurrence

## Abstract

This study aimed to determine whether endocervical glandular involvement (GI) affects the clinical prognosis of patients with cervical intraepithelial neoplasia (CIN) III who underwent the loop electrosurgical excision procedure (LEEP). This retrospective study included 250 patients who underwent LEEP for the treatment of CIN III between August 2005 and May 2020. The medical records of 234 patients were analyzed; 137 (58.5%) patients were GI negative, and 97 (41.5%) were GI positive. Margin involvement of the LEEP specimen was found in 59 (45.4%) patients in the GI-negative group and 54 (58.7%) patients in the GI-positive group (*p* = 0.051). The additional surgical procedures (repeat conization or hysterectomy) were significantly more performed in GI-positive patients than in GI-negative patients (40.9% vs. 23.1%, *p* = 0.004). When comparing the LEEP specimens of GI-1 (GI-positive confirmed via cervical biopsy before conization) and GI-2 (GI-positive confirmed via conization), we found that the mean depth was significantly greater in the GI-1 group (10.9 mm) than in the GI-2 group (7.6 mm) (*p* = 0.024). Surgical margin involvement was more frequently observed in the GI-2 group than in the GI-1 group (*p* = 0.030). There was no significant difference in the recurrence rates of CIN between the GI-negative and GI-positive groups (*p* = 0.641). In conclusion, despite no significant differences in residual disease and CIN recurrence between the GI-negative and GI-positive groups, additional surgical treatments were more frequently performed in GI-positive patients. Repeat surgery based on GI positivity should be carefully considered to avoid overtreatment and surgical complications.

## 1. Introduction

Cervical intraepithelial tumors (CIN) are precursors of cervical cancer that can progress to malignant lesions unless treated appropriately [1]. The recommended treatment for CIN II+ (CIN II and III) lesions is conization, followed by routine human papillomavirus (HPV) and/or cervical cytology tests [2]. The suggested risk factors for recurrence after conization are age, margin involvement (MI), high-grade CIN, persistent HPV infection, and glandular involvement (GI) [3,4,5,6,7,8]. Glandular involvement (GI) is defined as the presence of squamous intraepithelial lesions in pre-existing glandular structures [9]. It is associated with high-risk HPV infections, high-grade CIN, and MI [9,10]. Previous studies have shown that GI positivity is associated with CIN recurrence regardless of margin status [11]. In addition, GI-positive conization specimens are associated with an increase in the frequency of subsequent surgical treatments [12]. However, another study that examined patients who underwent hysterectomy within 6 months of conization reported that the residual lesion was not different between GI-positive and GI-negative patients [13]. It is important to properly understand the clinical effects of GI on disease prognosis. The clinical significance of GI has not been widely studied. Therefore, in this study, clinical prognosis according to GI status was investigated in patients who underwent conization for treatment of CIN III. In addition, we evaluated the effects of GI status on the clinical course of patients with CIN III.

## 2. Methods

This retrospective study included patients whose conditions were histologically diagnosed as CIN III between August 2005 and May 2020 at a tertiary medical center. After obtaining Institutional Review Board approval (No. KUMC 2021-07-021), the following data of patients were collected: clinical characteristics; HPV infection status; and histopathologic results, including depth, margin status, and glandular involvement of the conization specimen. Patients diagnosed with invasive cervical cancer before conization or incomplete pathologic records were excluded.

Conization was performed using a loop electrosurgical excision procedure (LEEP). There are two types of conization: a single-layer conization that sweeps the cervix only once, and a two-layer conization that adds a smaller loop endocervical canal resection to exocervical conization. The type of procedure to be performed was at the physician’s discretion. In the case of two-layer conization, the sum of the two specimen depths was used as the conization depth. MI was assessed separately in three areas (endocervical, exocervical, and deep margins), and CIN involvement at any of these sites was considered margin-positive. GI status was determined via a cervical biopsy or conization. In this study, GI-1 was defined as GI-positive as confirmed via cervical biopsy before conization, whereas GI-2 was defined as GI-positive as confirmed via conization. Clinical outcomes of the two groups were compared. Patients who underwent conization were followed up with cervical cytology and/or HPV testing every 3–6 months. Persistent HPV infection was defined as HPV infection detected on two consecutive tests. 

Data analyses were performed using SPSS for Windows (version 17; SPSS Inc., Chicago, IL, USA). Categorical variables are presented as numbers and percentages. Continuous variables were analyzed using Student’s *t*-test or Mann–Whitney test according to the results of the Kolmogorov–Smirnov test for the confirmation of normal distribution. Categorical variables were analyzed using the chi-square test and Fisher’s exact test. Statistical significance was set at *p* < 0.05.

## 3. Results

A total of 234 patients were examined during the study period. The clinical characteristics of the study population are shown in Table 1. The mean age was 42.3 years (range, 22–77 years), with 53 (23.3%) patients being postmenopausal. Single-layer conization was performed for 125 (56.8%) patients and two-layer conization for 95 (43.2%) patients. MI was observed in 113 patients (50.9%). The number of GI-negative and GI-positive patients was 137 (58.5%) and 97(41.5%), respectively. 

**Figure 1:**
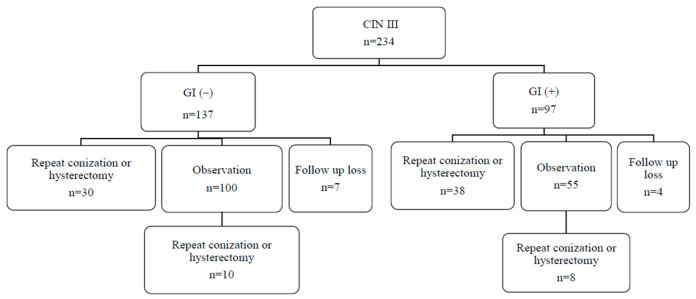
Clinical management according to the glandular involvement status.

Clinical management according to GI status is shown in Figure 1. Thirty (21.9%) patients in the GI-negative group and 38 (39.2%) patients in the GI-positive group underwent repeat conization or hysterectomy within 6 months of initial conization; 100 (73.0%) patients in the GI-negative group and 55 (56.7%) patients in the GI-positive group were routinely followed up. During the follow-up period, 10 patients in the GI-negative group and 8 patients in the GI-positive group underwent repeat conization or hysterectomy. 

Treatment outcomes were compared between the GI-negative and GI-positive groups (Table 2). There were no significant differences in age, parity, menopause, high-risk HPV infection, and persistent HPV infection. Although surgical MI was not significantly different (*p* = 0.051), repeat conization or hysterectomy within 6 months was performed significantly more in the GI-positive group (*p* = 0.004). 

Among 97 GI-positive patients, the condition of 34 was diagnosed via cervical biopsy prior to conization (GI-1) and that of 63 via conization (GI-2). The treatment outcomes of GI-positive patients were compared between GI-1 and GI-2 (Table 3). Deep conization was more frequently performed in GI-1 than in GI-2 (*p* = 0.024). Surgical MI was lower in GI-1 than in GI-2 (*p* = 0.030). Repeat conization or hysterectomy was performed less frequently in GI-1 than in GI-2 (27.3% vs. 48.3%, *p* = 0.048).

The prognosis of CIN III patients according to clinical management is shown in Table 4. Sixty-eight patients underwent repeat conization or hysterectomy approximately 4 weeks after initial conization. There was no significant difference in residual cervical disease between GI-negative and GI-positive patients (*p* = 0.954). Three cases were diagnosed as invasive cervical cancer in the repeat conization or hysterectomy group. Of the 155 patients observed after initial conization, 18 underwent repeat conization or hysterectomy. The mean period between the additional procedure and initial conization was 23.3 months. There was no significant difference in recurrent disease between GI-negative and GI-positive patients (*p* = 0.641) in the observation group. One case was diagnosed as invasive cervical cancer approximately 4 years after the initial conization in the observation group. The patient was GI- and MI-negative at the initial conization.

## 4. Discussion

This study found that the prognosis of CIN III patients who underwent LEEP was not different between the GI-positive and GI-negative groups. However, regardless of MI status, additional surgical treatment after conization was more frequently performed for GI-positive patients than for GI-negative patients. In addition, patients who were identified as GI-positive via cervical biopsy before conization underwent deep conization and showed a relatively low MI rate.

A GI-positive diagnosis before conization could help in the adequate treatment of CIN. GI-positive lesions have a four-fold increased association with high-grade lesions than with low-grade lesions [14,15,16]. Therefore, GI positivity may suggest the diagnosis in favor of HSIL. In the present study, GI positivity was confirmed via cervical biopsy or conization, and the detection rate with cervical biopsy was 35.1%. This rate, however, had limitations because it was of a spot biopsy rather than excision of the entire cervical lesion. According to Anderson et al., the median depth of the involved gland was 1.24 mm and a depth of 2.92 mm could detect 95% of the involved gland [17]. Therefore, an adequate depth of cervical biopsy could increase the early detection of GI.

Previous studies have demonstrated that GI status is associated with disease prognosis after conization. Demopoulos et al. reported that MI and GI positivity were significantly associated with the recurrence of CIN III in patients who underwent conization [7]. Other studies have reported that GI status is significantly predictive of residual CIN [18] and that the cumulative rate of recurrence is significantly higher in GI-positive patients [6,12]. However, GI positivity was not considered a risk factor for recurrent and residual cervical lesions in this study. Both the recurrent disease in the observation group and residual lesions in the additional treatment group did not differ according to GI status. Similarly to these results, Moore et al. reported that GI positivity was not a significant factor associated with residual lesions within 1 year after conization [19]. Another study showed that there was no relationship between GI positivity and residual lesions in a multivariable logistic regression analysis (*p* = 0.575) [20].

GI-positive patients are likely to receive additional treatment owing to concerns about residual disease. Regardless of MI status, repeat conization or hysterectomy after initial conization was performed significantly more often in the GI-positive group than in the GI-negative group in this study. MI is widely accepted as a risk factor for recurrence [21,22]. Patients with MI have a 5-fold higher relative risk of persistent or recurrent CIN II+ lesions than margin-negative patients [2]. Therefore, repeat conization or hysterectomy is considered appropriate for patients with MI who have no concerns about future fertility [22,23,24]. However, unlike MI, there is insufficient evidence for repeat conization or hysterectomy in GI-positive patients.

Concerning GI-1 and GI-2 positivity, the mean depth of conization was significantly different between the two groups (11 mm vs. 8 mm, *p* = 0.024). In addition, MI was significantly lower in GI-1 than in GI-2 (44.1% vs. 67.2%, *p* = 0.03). These results correlate with those of previous studies that demonstrated that deeper conization reduces MI [25,26,27]. Although MI can decrease with increasing depth of conization, deep conization is associated with adverse obstetric outcomes, postoperative bleeding and cervical stenosis [28]. Many studies have been conducted on determining the appropriate depth of conization. A study suggested a conization depth cut-off value of 18 mm to avoid endocervical MI [26]. In another study, a conization depth of 8 mm was adequate when the squamocolumnar junction was fully visible in the exocervix, and a conization depth of 15–20 mm was adequate when the squamocolumnar junction was not fully visible [29]. However, Lara-Peñaranda et al. reported that a conization depth < 10 mm did not increase disease persistence [30]. In our study population, the mean conization depth was 8.6 mm and the overall recurrence rate was 7.7%. Despite the relatively thin depth of conization in our study, the recurrence rate was consistent with previous studies that reported a recurrence rate of 6–9% [4,31]. In the present study, the depth of conization in GI-2 was thinner than that in GI-1, which was related to increased MI rates and additional surgical procedures. Therefore, to reduce MI and additional surgical procedures, it is important to perform conization at an appropriate depth.

In conclusion, there were no differences in residual disease and CIN recurrence between the GI-negative and GI-positive groups of CIN III patients who underwent LEEP. However, additional surgical treatments were more frequently performed for GI-positive patients regardless of MI. Repeat surgery for only GI-positive patients without MI should be carefully considered to avoid overtreatment and surgical complications, such as adverse obstetric outcomes and surgical morbidity. Further studies on the appropriate depth of conization and follow-up management are needed.

## Figures and Tables

**Table 1:** Clinical characteristics of the study population (*n* = 234).

Categories	Number	%
Age (year)	42.3 ± 12.2	
Parity	1.5 ± 1.2	
Menopause		
No	174	76.7
Yes	53	23.3
High-risk HPV infection		
No	7	3.8
Yes	179	96.2
Conization depth (mm)	8.6 ± 4.6	
Margin involvement		
No	109	49.1
Yes	113	50.9
Glandular involvement		
No	137	58.5
Yes	97	41.5
Follow-up duration (months)	45.2 ± 38.4	

**Table 2:** Comparison of clinical factors and treatment outcomes between glandular involvement (GI)-negative and GI-positive patients.

	GI-Negative Group (*n* = 137)	GI-Positive Group (*n* = 97)	*p*-Value
Age (year)	43.4 ± 12.0	40.8 ± 12.3	0.065
Parity			
No	26 (21.7)	27 (30.7)	0.140
Yes	94 (78.3)	61 (69.3)	
Menopause			
No	100 (75.8)	74 (77.9)	0.707
Yes	32 (24.2)	21 (22.1)	
High-risk HPV infection			
No	3 (2.8)	4 (5.1)	0.460
Yes	104 (97.2)	75 (94.9)	
Persistent HPV infection			
No	84 (83.2)	50 (78.1)	0.419
Yes	17 (16.8)	14 (21.9)	
Margin involvement			
No	71 (54.6)	38 (41.3)	0.051
Yes	59 (45.4)	54 (58.7)	
Repeat conization or hysterectomy			
No	100 (76.9)	55 (59.1)	0.004
Yes	30 (23.1)	38 (40.9)	

GI: Glandular involvement; HPV: Human papillomavirus.

**Table 3:** Comparison of treatment outcomes between glandular involvement (GI)-1 and GI-2 among GI-positive patients (*n* = 97).

	GI-1 (*n* = 34)	GI-2 (*n* = 63)	*p*-Value
Conization depth (mm)	10.9 ± 6.8	7.6 ± 3.2	0.024
Conization width (mm)	27.2 ± 6.6	24.9 ± 11.1	0.023
Margin involvement			
No	19 (55.9)	19 (32.8)	0.030
Yes	15 (44.1)	39 (67.2)	
Repeat conization or hysterectomy			
No	24 (72.7)	31 (51.7)	0.048
Yes	9 (27.3)	29 (48.3)	

GI: glandular involvement; GI-1: GI (+) diagnosed by cervical biopsy; GI-2: GI (+) diagnosed by conization.

**Table 4:** Prognosis of clinical management according to the glandular involvement status.

	Repeat Conization or Hysterectomy (*n* = 68)			Observation (*n* = 155)		
	GI (−)	GI (+)	*p*-Value	GI (−)	GI (+)	*p*-Value
Non-specific finding	16 (53.3)	20 (52.6)	0.954	93 (93.0)	50 (90.9)	0.641
Residual or recurrent disease	14 (46.7)	18 (47.4)		7 (7.0)	5 (9.1)	
CIN I	2	6		2	1	
CIN III	10	11		4	4	
Invasive cancer	2	1		1	0	

GI: glandular involvement; CIN: cervical intraepithelial neoplasia.

## Data Availability

The datasets use and/or analyzed during the current study will be provided by the corresponding author on reasonable request.
